# Fine particulate air pollution and its components in association with cause-specific emergency admissions

**DOI:** 10.1186/1476-069X-8-58

**Published:** 2009-12-21

**Authors:** Antonella Zanobetti, Meredith Franklin, Petros Koutrakis, Joel Schwartz

**Affiliations:** 1 Department of Environmental Health, School of Public Health, Harvard University, Boston MA, USA; 2 Department of Statistics, University of Chicago, Chicago IL, USA

## Abstract

**Background:**

Although the association between exposure to particulate matter and health is well established, there remains uncertainty as to whether certain chemical components are more harmful than others. We explored whether the association between cause-specific hospital admissions and PM_2.5 _was modified by PM_2.5 _chemical composition.

**Methods:**

We estimated the association between daily PM_2.5 _and emergency hospital admissions for cardiac causes (CVD), myocardial infarction (MI), congestive heart failure (CHF), respiratory disease, and diabetes in 26 US communities, for the years 2000-2003. Using meta-regression, we examined how this association was modified by season- and community-specific PM_2.5 _composition, controlling for seasonal temperature as a surrogate for ventilation.

**Results:**

For a 10 μg/m^3 ^increase in 2-day averaged PM_2.5 _concentration we found an increase of 1.89% (95% CI: 1.34- 2.45) in CVD, 2.25% (95% CI: 1.10- 3.42) in MI, 1.85% (95% CI: 1.19- 2.51) in CHF, 2.74% (95% CI: 1.30- 4.2) in diabetes, and 2.07% (95% CI: 1.20- 2.95) in respiratory admissions. The association between PM_2.5 _and CVD admissions was significantly modified when the mass was high in Br, Cr, Ni, and Na^+^, while mass high in As, Cr, Mn, OC, Ni, and Na^+ ^modified MI, and mass high in As, OC, and SO_4_^2- ^modified diabetes admissions. For these species, an interquartile range increase in their relative proportion was associated with a 1-2% additional increase in daily admissions per 10 μg/m^3 ^increase in mass.

**Conclusions:**

We found that PM_2.5 _mass higher in Ni, As, and Cr, as well as Br and OC significantly increased its effect on hospital admissions. This result suggests that particles from industrial combustion sources and traffic may, on average, have greater toxicity.

## Background

Many studies have shown that ambient particulate air pollution (PM), generally measured as particles with aerodynamic diameter less then 10 micrometers (PM_10_), is associated with increased risk of hospital admissions for broadly defined cardiovascular or respiratory causes [1-6]. Similar relationships have been reported in locations reflecting a wide range of particle composition, and concentrations of gaseous co-pollutants [7-11].

Diabetics are more susceptible to particles and previous panel studies and time series analyses have shown this [12-14].

While previous studies have primarily used ambient PM_10 _as an exposure metric, PM_2.5 _(particles with aerodynamic diameter less then 2.5 micrometers) have become a greater health and regulatory concern due to epidemiologic studies suggesting that PM_2.5 _might have greater toxicity than larger particles [15,16].

Nevertheless, as the US Environmental Protection Agency (US EPA) did not begin monitoring PM_2.5 _concentrations until 1999, the literature on the association between PM_2.5 _and mortality [16-21] is relatively sparse. There have been even fewer studies examining the association of PM_2.5 _with hospital admissions [22-25].

Fine particles consist of a large number of compounds and their composition varies spatially and temporally. Its components can be associated with specific sources, such as elemental carbon with traffic, nickel with oil burning and selenium with coal burning power plants. While the evidence for the health effects of fine particles has been growing, there is uncertainty as to which components of these particles are most harmful. Moreover, a better understanding of the relative toxicity of particles with differing chemical composition will in turn lead to more targeted emission control strategies and regulations.

One main limitation in examining PM_2.5 _components is the irregularity of the PM_2.5 _speciation data available from the EPA Speciation Trends Network (STN) monitoring sites. The STN was established in 2000 and monitors only report data for every third or sixth day, thus limiting the statistical power necessary to detect associations between individual species and health events. The lack of daily sampling also prevents the examination of time lags and effect of multi-day exposures.

Consequently, there have been few studies to date examining the health effects associated with PM_2.5 _components. One study [26] examined the associations between 19 PM_2.5 _components and daily mortality in six California counties, and found that PM_2.5 _mass and several constituents (EC, OC, NO_3_^-^, Fe, K, Ti) were associated especially with cardiovascular deaths at various lags. Another study [27] used the elemental composition of PM_2.5 _to investigate the effect of traffic, residual oil and power plant emissions on daily mortality in six US cities. Their results indicate that combustion particles in the fine particles from mobile and coal combustion sources were associated with increased mortality.

The most recent study [28] examined the differential effects of PM_2.5 _species on mortality in 25 U.S. communities. This study differs from the two previous studies, in that rather than directly using the observed particle component concentrations in their main model, they overcame the issue of limited statistical power and inability to examine more than one day exposure due to poor temporal coverage of data reported from the STN sites by using a hierarchical approach. The authors first determined the association between PM_2.5 _mass and mortality, and then in a second stage of the analysis, a meta-regression was used to examine how the pooled association was modified by community and season particle composition. They found evidence that Ni (predominantly from oil combustion) as well as Sulfate and As (from coal burning power plants) increased the mortality risk associated with PM_2.5_.

In this study we applied the method of Franklin and co-authors to examine the association between cause-specific hospital admissions and PM_2.5 _in 26 U.S. communities, and explore whether PM_2.5 _chemical composition played a role in its toxicity.

## Methods

### Air Pollution and Meteorological Data

The PM_2.5 _mass and species concentration data were obtained online from the EPA Technology Transfer Network Air Quality System [29].

We selected the same cities studied by Franklin et al [30], but also included Chicago, IL, as we had MEDICARE and sufficient speciation data between 2000 and 2003. These cities were originally chosen due to availability of daily PM_2.5 _data. For most of these cities, the metropolitan county encompassed the city and much of its suburbs, but we used multiple counties for Boston (Suffolk, Norfolk, and Middlesex), and Minneapolis-St. Paul (Ramsey and Hennepin). Henceforth we refer to the analyzed geographical areas as communities.

The STN monitors operate on a 24 hour schedule and collect particles on Teflon, nylon or quartz filters which are analyzed for trace elements using X-ray fluorescence, for ions using ion chromatography and for organic and elemental carbon using thermal-optical analysis.

The EPA maintains multiple PM_2.5 _mass sites, but typically only one PM_2.5 _speciation site within a county. In order to use all the available PM_2.5 _monitoring sites, the 24-hour integrated mass concentrations were averaged over the county using a method previously described [31]. Briefly, we computed local daily mean concentrations using an algorithm that accounts for the different monitor-specific means and variances. However, before averaging, any monitor that was not well correlated with the others (r < 0.8 for two or more monitor pairs within a community) was excluded as it likely measured a local pollution source and would not represent the general population exposure over the entire county. The number of monitors across the counties varied between 1 and 4.

Based on results from previous epidemiological studies [26-33] we focused on the species with different sources and toxicological background. In particular we focus on the paper of Franklin et al [30] who also screened the STN data for inconsistencies based on the percentage of data below the minimum detection limit and with quality control flags. We therefore examined the following species: Arsenic (As), Aluminium (Al), Bromine (Br), Chromium (Cr), Iron (Fe), Lead (Pb), Manganese (Mn), Nickel (Ni), Potassium (K), Silicon (Si), Vanadium (V), Zinc (Zn), ions nitrate (NO_3_^-^), Sulfate (SO_4_^2-^), ammonium (NH_4_^+^), sodium (Na^+^), elemental carbon (EC) and organic carbon (OC).

For all available observations we computed the ratio between each species and PM_2.5 _mass and then took averages by season across all years to obtain season- and community-specific long-term mean seasonal concentration ratios.

Meteorological data including daily mean temperature and dew point temperature from the predominant weather station in each community were acquired from the National Climatic Data Center [34].

### Health Data

We extracted data on emergency hospital admissions from the Health Care Financing Administration (MEDICARE) billing records for the years 2000-2003. The MEDICARE system provides hospital coverage for all US citizens aged 65 and over.

Based on evidence from previous studies we chose to examine causes of admissions which have been associated with particulate matter and added diabetes as it is related to CVD complications. We defined cases as persons admitted from the emergency room with a primary discharge diagnosis of: myocardial infarction (MI, International Classification of Disease ninth revision (ICD-9): 410), diabetes (ICD-9: 250), congestive heart failure (CHF, ICD-9:428), cardiac disease (CVD, ICD-9:390-429), and all respiratory disease (RESP, ICD-9:460-519).

### Statistical Methods

We applied a time series analysis using Poisson regression to examine the association between daily counts of cause-specific admissions and daily PM_2.5 _mass concentrations in each community. In each community, the analysis was stratified by season, since the composition of particles varies seasonally, due in part to different source contributions at different times of the year. In each community, we controlled for season and long term trend with a natural cubic regression spline with 1.5 degrees of freedom (d.f.) for each season and year (corresponding to six d.f. per year); day of the week using indicator variables; and three-day averaged temperature and dew point temperature with a natural cubic spline with three d.f..

The effect estimates were expressed as a percent increase in hospital admission with a 10 μg/m^3 ^increase in PM_2.5 _mass concentration averaged over the day before and the day of admission.

In the second stage of the analysis, we combined the Poisson regression effect estimates using random effects meta-analysis [35] to obtain an overall effect across all the communities. The season and community specific long-term mean seasonal concentration ratios, which reflect particle composition and thus the relative contribution of different sources to the PM_2.5 _mass, were then used in a meta-regression to quantify to what extent the association between PM_2.5 _mass and admissions was modified by particle composition. This involved regressing the community and season-specific Poisson estimates (four for each community for a total of 104 coefficients) against the community and season-specific mean concentration ratios; we first included one element at the time and then we included those species that were significant effect modifiers in that first stage in a multivariate model. In the meta-regression, the variance was composed of the sum of estimated variance from the first stage, and a random variance-covariance matrix component reflecting heterogeneity over and above what can be explained by the modifier variables, as previously described by Franklin et al [28] and Zanobetti et al [36].

Franklin et al [30] also found that the association between PM_2.5 _mass and mortality was modified by the seasonal average temperature and used it as a surrogate to explain ventilation of ambient air to the indoor environment [37]. They showed an inverted U-shape relationship with the PM_2.5 _- mortality effect estimates and temperature indicating that at extremes of temperature, when windows and doors are closed resulting in reduced ventilation, the effect of ambient PM_2.5 _on mortality was smaller than at moderate temperatures. We assumed that this phenomenon held true for hospital admissions and thus the meta-regression for each outcome included a linear and quadratic term to control for mean temperature.

We used the I^2 ^statistic to assess the proportion of total variation in effect estimates that was due to between-community heterogeneity [38]. The I^2 ^statistic, I^2 ^= [Q/(k - 1)] - 1/[Q/(k - 1)] where k is the number of communities, is a generalization of the Χ^2 ^or Q test for heterogeneity and expresses the proportion of variance explained. When Q/(k - 1) is below 1 there is no heterogeneous variability in the estimates.

Finally, as there is evidence that socioeconomic status (SES) plays a role in the health effects of particles (Finkelstein 2003; Levy 2000), we examined community-specific parameters including: median household income, percent of population below poverty line, percent of adult population having graduated high school, and percent of all households having the head of the household 65 years of age or older and below poverty level in 1999, obtained from the US Census Bureau [39]. The community-specific prevalence of central air conditioning (AC), obtained from the American Housing Survey [40], was also examined to address the potential for any residual heterogeneity associated with ventilation/particle penetration not sufficiently accounted for with the quadratic term for seasonally averaged temperature. Each parameter was included separately in the meta-regression along with temperature and each species proportion. Although SES parameters and central AC prevalence did not vary seasonally, both were included in the seasonal meta-regression.

We used SAS 9.1 [41] for data management, and R 2.7.2 [42] for regression modelling.

## Results

Table 1 shows the mean number and standard deviation of daily hospital admissions in each community by cause, together with the distribution of the 2-day moving average of PM_2.5_. Over all 26 communities we examined 685,716 CVD, 121,652 MI, 238,587 CHF, 46,192 diabetes, and 261,449 respiratory related admissions.

**Table 1:** Community specific mean and standard deviation for the analyzed causes of hospital admissions for citizen 65 years and older during the years 2000-2003, and for PM_2.5 _averaged over two days.

	CHF		CVD		MI		Diabetes		Respiratory		PM_2.5_	
	
City	mean	std	mean	std	mean	std	mean	std	mean	std	mean	std
Akron, OH	4.0	2.2	11.4	3.9	1.9	1.4	0.6	0.7	8.2	3.6	16.2	7.5
Bakersfield, CA	1.1	1.0	3.0	1.7	0.6	0.8	0.2	0.5	1.2	1.1	21.0	17.1
Boston, MA	13.2	4.1	36.3	7.5	6.2	2.6	2.1	1.5	13.9	4.6	13.4	5.9
Chicago, IL	29.2	7.0	81.2	14.0	13.7	3.9	6.0	2.7	27.6	7.1	16.1	7.4
Cleveland, OH	10.0	3.6	27.6	6.5	4.4	2.1	1.6	1.3	9.9	3.8	16.9	8.0
Columbus, OH	4.4	2.3	12.1	3.9	1.9	1.4	0.6	0.8	4.6	2.4	16.6	7.5
Dallas, TX	6.0	2.7	17.1	4.9	3.2	1.8	1.2	1.1	6.5	3.0	12.8	5.6
Dayton, OH	3.1	1.8	8.6	3.1	1.4	1.2	0.5	0.7	4.0	2.2	16.2	7.5
Detroit, MI	14.2	4.3	42.4	8.1	7.4	2.8	3.0	1.7	13.8	4.4	16.2	8.1
El Paso, TX	2.7	1.8	8.7	3.4	1.3	1.2	1.3	1.2	6.5	3.5	10.2	4.8
Erie, PA	1.3	1.2	4.0	2.1	0.7	0.9	0.2	0.4	1.2	1.1	13.3	7.2
Fresno, CA	2.3	1.6	7.0	2.8	1.5	1.2	0.5	0.7	2.1	1.6	20.0	15.6
Harrisburg, PA	1.2	1.2	3.7	2.0	0.8	0.9	0.2	0.4	1.0	1.0	15.6	8.3
Houston, TX	8.8	3.4	23.9	6.3	3.9	2.1	1.9	1.5	9.0	3.7	12.8	5.2
Kansas City, MO	2.2	1.5	6.5	2.8	1.2	1.1	0.3	0.6	2.5	1.7	12.7	5.7
Los Angeles, CA	12.5	4.2	40.5	8.0	7.6	2.9	3.0	1.8	16.2	4.8	20.7	11.2
Minneapolis, MN	3.8	2.0	13.2	3.9	2.8	1.7	0.7	0.8	4.7	2.3	10.6	5.9
Philadelphia, PA	10.1	3.6	24.7	6.4	3.3	1.9	2.1	1.4	9.6	3.6	15.0	8.0
Pittsburgh, PA	9.3	3.4	24.0	6.2	3.9	2.1	1.4	1.2	9.5	3.6	15.5	7.8
Port Arthutr, TX	1.4	1.2	4.2	2.1	0.7	0.8	0.3	0.6	1.5	1.3	11.4	5.5
Riverside, CA	1.6	1.2	5.5	2.4	1.2	1.1	0.3	0.6	1.9	1.4	27.4	15.5
Sacramento, CA	1.1	1.1	3.7	2.0	0.9	0.9	0.2	0.5	1.5	1.3	12.5	10.2
San Diego, CA	3.0	1.8	10.0	3.5	2.3	1.6	0.6	0.8	3.8	2.1	15.2	8.4
Seattle, WA	3.0	1.8	9.8	3.2	2.2	1.5	0.4	0.6	4.3	2.3	10.0	5.6
St. Louis, MO	6.6	2.6	18.8	4.6	3.9	2.0	1.1	1.1	7.3	3.1	15.0	6.8
Toledo, OH	2.4	1.5	6.7	2.7	1.3	1.2	0.4	0.6	2.5	1.7	14.9	7.3
												
Overall	6.1	2.5	17.5	4.5	3.1	1.7	1.2	1.0	6.7	2.8	15.3	8.2

When looking across all seasons (Table 1), El Paso, TX, Minneapolis, MN, and Seattle, WA, had the lowest PM_2.5 _concentrations, while Riverside, Bakersfield, and Los Angeles, CA had the highest concentrations. The lowest mean concentration of PM_2.5 _mass, when looking by season (not shown), was observed in Sacramento in spring (6.1 μg/m^3^), well below the National Ambient Air Quality Standard (NAAQS) of 15 μg/m^3^, while the highest springtime concentration was in Riverside (24 μg/m^3^). The highest winter values were observed in Bakersfield and Fresno, CA, which were 29.5 and 29.9 μg/m^3^, respectively.

**Figure 1:**
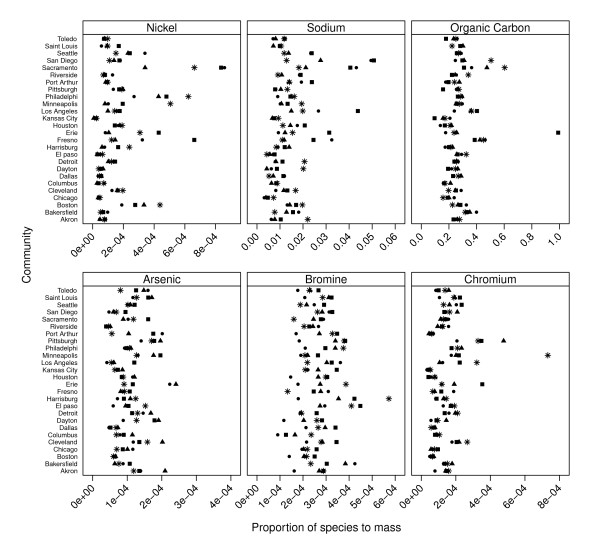
**Community and season-specific proportions of species to PM_2.5 _mass for 6 selected species: Arsenic, Bromine, Chromium, Organic Carbon, Sodium, and Nickel**. The seasons are: "black triangle" = winter; "black square" = spring; "star" = summer; "black circle" = autumn.

Species to PM_2.5 _mass proportions averaged over all communities are presented in Table 2, while community and season-specific proportions of six selected species are illustrated in Figure 1. Notably, organic carbon was higher in all Californian communities in all seasons compared to other communities; while Ni was higher in Sacramento, Philadelphia, Minneapolis, and Boston in winter and in Harrisburg and Fresno in spring.

**Table 2:** Distribution of the community and season averaged species - to-PM_2.5 _mass proportions for selected species, across all communities.

	Min.	1st Qu.	Median	Mean	3rd Qu.	Max.
Winter						
As	0.00005	0.00007	0.00009	0.00010	0.00013	0.00017
Br	0.00013	0.00021	0.00025	0.00027	0.00030	0.00057
Cr	0.00005	0.00008	0.00013	0.00017	0.00019	0.00073
EC	0.02800	0.04440	0.05921	0.06179	0.07297	0.11720
Mn	0.00007	0.00012	0.00020	0.00025	0.00029	0.00076
Na^+^	0.00428	0.00916	0.01234	0.01326	0.01784	0.02206
Ni	0.00002	0.00007	0.00012	0.00018	0.00020	0.00066
OC	0.15770	0.22330	0.26060	0.28270	0.30940	0.60360
SO_4_^2-^	0.05094	0.08475	0.18840	0.17280	0.23510	0.28830
Spring						
As	0.00004	0.00009	0.00011	0.00012	0.00013	0.00020
Br	0.00013	0.00027	0.00030	0.00033	0.00034	0.00110
Cr	0.00004	0.00009	0.00013	0.00015	0.00020	0.00035
EC	0.02337	0.04498	0.05780	0.06021	0.06758	0.14310
Mn	0.00008	0.00016	0.00021	0.00037	0.00035	0.00260
Na^+^	0.00471	0.01003	0.01325	0.01783	0.02300	0.05077
Ni	0.00002	0.00007	0.00015	0.00020	0.00019	0.00084
OC	0.09634	0.19490	0.24540	0.27130	0.28280	0.99030
SO_4_^2-^	0.12100	0.20270	0.25690	0.27600	0.30840	0.93540
Summer						
As	0.00004	0.00007	0.00010	0.00010	0.00013	0.00022
Br	0.00009	0.00018	0.00020	0.00022	0.00026	0.00043
Cr	0.00003	0.00007	0.00012	0.00012	0.00016	0.00021
EC	0.02345	0.03834	0.04898	0.04779	0.05715	0.08223
Mn	0.00010	0.00014	0.00018	0.00025	0.00029	0.00094
Na^+^	0.00315	0.00801	0.00952	0.01502	0.01838	0.04950
Ni	0.00002	0.00006	0.00010	0.00015	0.00017	0.00086
OC	0.13800	0.22310	0.25400	0.26160	0.29090	0.46250
SO_4_^2-^	0.17060	0.23030	0.29560	0.28970	0.33640	0.41870
Autumn						
As	0.00005	0.00008	0.00011	0.00012	0.00017	0.00024
Br	0.00017	0.00022	0.00027	0.00027	0.00029	0.00038
Cr	0.00004	0.00008	0.00014	0.00015	0.00019	0.00048
EC	0.03039	0.05546	0.06364	0.06337	0.07394	0.09023
Mn	0.00011	0.00017	0.00024	0.00030	0.00032	0.00088
Na^+^	0.00388	0.00727	0.01119	0.01131	0.01398	0.02762
Ni	0.00001	0.00006	0.00009	0.00013	0.00016	0.00043
OC	0.15760	0.24590	0.26240	0.27620	0.29270	0.47500
SO_4_^2-^	0.08987	0.16770	0.24910	0.22490	0.27560	0.33880

The meta-analysis results are shown in Table 3. Across all the seasons, for a 10 μg/m^3 ^increase in two-day averaged PM_2.5 _concentration we found a 1.89% increase (95% CI: 1.34-2.45) in CVD admissions, a 2.25% increase (95% CI: 1.10-3.42) in MI admissions, a 1.85% increase (95% CI: 1.19-2.51) in CHF admissions, a 2.74% increase (95% CI: 1.30-4.2) in diabetes admissions, and a 2.07% increase (95% CI: 1.20-2.95) in respiratory admissions. Seasonally, the percent increase in each cause of admission was found to be highest in the spring, while it was generally lower in summer and autumn, except for diabetes which was high in autumn as well.

**Table 3:** Estimated percent increase in hospital admissions for a 10 μg/m^3 ^increase in 2-day averaged PM_2.5 _by cause of admission and season

	%	95% CI	
Cardiovascular disease			
All seasons	1.89	1.34	2.45
Winter	2.60	1.60	3.60
Spring	3.38	2.47	4.30
Summer	0.13	-0.78	1.06
Autumn	1.49	0.49	2.50
			
Myocardial Infarction			
All seasons	2.25	1.10	3.42
Winter	2.10	0.42	3.81
Spring	4.50	1.42	7.68
Summer	2.09	-1.29	5.60
Autumn	0.68	-0.85	2.24
			
Congestive heart failure			
All seasons	1.85	1.19	2.51
Winter	2.90	1.63	4.19
Spring	4.14	2.61	5.68
Summer	0.11	-1.23	1.46
Autumn	0.80	-0.40	2.01
			
Diabetes			
All seasons	2.74	1.30	4.20
Winter	-0.52	-3.20	2.24
Spring	5.43	1.97	9.02
Summer	1.85	-1.02	4.80
Autumn	4.78	2.16	7.46
			
Respiratory disease			
All seasons	2.07	1.20	2.95
Winter	1.79	0.47	3.12
Spring	4.34	2.19	6.54
Summer	1.26	-0.60	3.16
Autumn	1.52	-0.06	3.13

We found significant though moderate heterogeneity among the community-specific effect estimates across all seasons (I^2^-statistic p < 0.05) for CVD and MI admissions, where 33% (CVD) and 24% (MI) of the total variability, as reported by the I^2 ^statistic, was attributable to between-community differences (as opposed to stochastic variation). For CHF, diabetes and respiratory admissions no significant heterogeneity among the community-specific effect estimates was found. We report the effect modification for all the causes analyzed even though cardiac effects were the only ones that displayed significant heterogeneity.

As we also found a similar inverted U-shaped association as shown in Franklin [28] between the effect estimates and season and city mean temperature, we adjusted for temperature with both a linear and square term in the meta-regression. Results of effect modification by species-to-mass proportions, adjusted for temperature, are shown in Table 4. For each species and cause of admission we present the P-value for the effect modification, and the percent increase for an IQR increase in the proportions with the 95% confidence interval (CI). We only show the results with a P-value < 0.07.

**Table 4:** Modification of the PM_2.5 _mass association across 26 US by PM_2.5 _composition.

		P-value for modifier	%	95% CI		IQR
Cardiovascular disease						
*Traffic*						
	Br	0.01	0.81	0.23	1.40	0.00010
*Sea salt, street salt*						
	Na^+^	< 0.01	0.87	0.35	1.39	0.00945
*Industrial combustion sources*						
	Ni	< 0.01	0.90	0.46	1.35	0.00012
	V	0.05	0.73	0.01	1.44	0.00017
*Soil and road dust*						
	Al	0.05	0.53	0.00	1.07	0.00193
Myocardial Infarction						
Traffic						
	OC	0.03	1.03	0.13	1.94	0.07060
*Industrial combustion sources*						
	Ni	0.04	1.13	0.04	2.22	0.00012
	As	< 0.01	2.35	0.84	3.85	0.00006
	Cr	0.05	1.34	0.00	2.68	0.00010
*Sea salt, street salt*						
	Na^+^	0.03	1.42	0.16	2.68	0.00945
*Soil and industrial sources*						
	Mn	0.03	0.99	0.14	1.85	0.00018
*Wood burning and soil*						
	K	0.05	1.61	0.01	3.22	0.00270
Diabetes						
*Traffic*						
	OC	< 0.01	-2.42	-3.79	-1.06	0.07060
	EC	0.02	-2.12	-3.84	-0.39	0.02469
*Industrial combustion sources*						
	As	0.04	2.16	0.11	4.21	0.00006
	SO_4_^2-^	0.01	2.91	0.92	4.89	0.11610
Congestive heart failure						
*Soil and road dust*						
	Al	0.07	0.77	-0.07	1.61	0.00193
*Industrial combustion sources*						
	Ni	0.07	0.64	-0.04	1.32	0.00012
Respiratory disease						
*Sea salt, street salt*						
	Na^+^	0.06	0.94	-0.02	1.91	0.00945
*Industrial combustion sources*						
	Ni	0.06	0.75	-0.03	1.53	0.00012

Results are expressed as % increase in cause-specific hospital admission per 10 μg/m^3 ^increase in PM_2.5 _for an IQR increase in the Species to PM_2.5 _mass proportions

We found that some species, Fe, NH_4_^+^, NO_3_^-^, Si, Zn, EC, and Pb did not result in any effect modification of the association between PM_2.5 _and hospital admissions.

Although single species are markers for more complex particle chemistry, for clarity of presentation we divided the species in the following groups to represent the general categories for which these elements are markers. Industrial combustion sources: Ni, V, As, Cr, SO_4_^2-^; Soil and road dust: Al; Traffic: Br, OC, EC; Sea salt, street salt: Na^+^; Soil and industrial sources: Mn; Wood burning and soil: K.

The association between mass and CVD admissions was significantly (p = 0.05) modified by Br, Ni, Na^+^, V and Al. Specifically, an IQR increase in the proportion of bromine in PM_2.5 _mass was associated with an additional 0.81% increase in CVD admissions (95% CI: 0.2-1.4). Similarly there was an additional 0.7% (95% CI: 0.01-1.44) increase for an IQR increase in V, a 0.9% increase (95% CI: 0.5-1.4) for an interquartile increase in Ni, and a 0.87% increase (95% CI: 0.4-1.4) for an IQR increase in Na^+ ^(Table 4). The association between PM_2.5 _and MI was modified by As, Cr, Mn, OC, Ni, K, and Na^+^.

Including a combination of species proportions that were statistically significant in Table 4 and performing a backward elimination, in multi-species models we found that for CVD the combination of Br and Ni remained statistically significant (P <= 0.05), with an additional 0.57% increase in CVD for an IQR increase in Br and an additional 0.80% increase for an IQR increase in Ni. When we examined MI, only Ni remained significant.

In a sensitivity analysis, changing the weather specification in the first stage to linear showed the same results as using cubic regression splines.

Central air conditioning (AC) and the SES variables were not statistically significant in the meta-regression and thus did not explain any residual heterogeneity in the effect estimates over and above what temperature and the species could explain. Moreover, the magnitude of the modification by the species proportions did not change drastically with the inclusion of AC or the SES parameters; they were only slightly increased or reduced.

**Figure 2:**
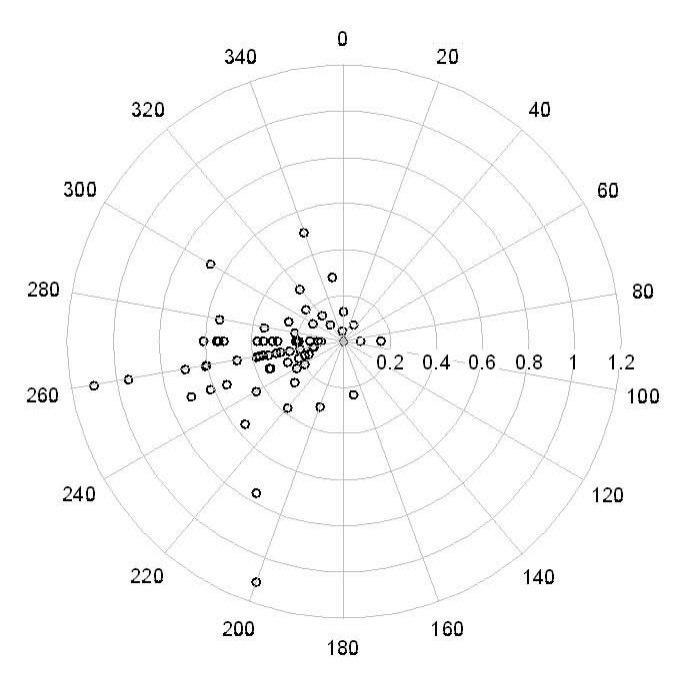
**Los Angeles: wind rose of the relationship between Na+ concentrations in ng/m^3 ^and wind direction**.

Because Na^+ ^and Ni were associated with both CVD and MI admissions, we examined their concentrations more carefully and found that levels were consistently higher in California. For Na^+^, this may be attributable to the contribution of marine aerosol from the Pacific Ocean. To support this we present the relationship between Na^+ ^concentrations and wind direction for the Los Angeles site. As shown by the wind rose (Figure 2), higher levels were observed when air masses passed over the sea (coming from SW). As a comparison, a similar wind rose was examined for EC, typically associated with local traffic (Figure 2), but no wind pattern was observed. Interestingly, the wind roses for Ni and V were similar to Na^+^, suggesting that wind coming over the ocean also carries elements often associated with ship emissions (not shown).

## Discussion

In this multi-community study we found a significant association between PM_2.5 _mass and both respiratory and cardiac hospital admissions. These effects were strongest in spring and were significantly modified by certain chemical components of the mass. The rationale for using species-to-mass concentration ratios in the second stage was that in the first stage the admission risk was estimated per unit of the total PM_2.5 _mass, which encompasses all measured species, and therefore effect modification by the species was best expressed on a per unit of PM_2.5 _mass basis.

One study [24] reported associations between hospital admissions and PM_2.5_, but the authors examined different categories of admissions than us, except for heart failure. They reported an association of 1.28% (95% CI, 0.78%-1.78%) increase in risk of heart failure per 10 μg/m^3 ^increase in same-day PM_2.5_, which is comparable to our estimate of 1.85% (95% CI: 1.19-2.51) increase in CHF for the same and previous day average of PM_2.5_. Similar results have been found by Peng [43], Metzger [44], and by Halonen [45]. Bell and co-authors [23] found higher effects in winter; they used a two-stage Bayesian hierarchical model which included two interaction terms allowing both the exposure and the temporal trend to differ by season. Instead we stratified by season, allowing a more specific control for season and trend within each community. Another difference is that the authors included cities with systematically missing data, which may have several implications in modelling the time series.

Two recent studies [22] examined the toxicity of PM_2.5 _chemical composition on hospital admissions. Bell et al [22] used the method we introduced in Franklin 2008 and used here; in the second stage however, they didn't take into account indoor infiltration. We had previously demonstrated [28] that mean seasonal temperature was an important predictor of the effect of PM_2.5 _in that city and season, and since species concentrations can vary with temperature, an important confounder of assessing the role of species. We controlled for this in the analyses in this paper. Bell examined a larger number of communities and utilized same-day (lag 0) PM_2.5 _concentrations versus the mean of lags 0 and 1 in our study. They found associations with Ni, EC and V while we did not see any effects for EC. Peng et al [46] used time-series analysis of the measured (one in three or one in six day) PM_2.5 _species, and found significant effects at lag 0 for EC, OC and ammonium, but not for Ni. Some of these differences may be due to confounding by ventilation patterns.

Our findings have several important parallels with Franklin and co-authors [28]. For instance, across all admission types, we found that the elements emitted primarily through industrial combustion, namely Ni and As, displayed the greatest and most consistent effect modification. We also found moderate modification by Al, which was shown to be a strong modifier in the previous study. The primary differences were that we found that Br and OC, species often associated with traffic, and Na^+^, an ion often associated with marine aerosol, were effect modifiers of several causes of admissions.

We also found a significant effect of PM_2.5 _on admissions for diabetes, which, despite the smaller daily counts, shows the strongest association in all season, spring, and autumn. As previous studies reported, diabetics might be particularly susceptible to particles [12-14]. We found that that SO_4_^2- ^and As were significant effect modifiers, associated with higher rates of diabetes admissions, whereas OC was associated with lower rates. This finding are consistent with that of O'Neill et al [13], who reported that SO_4_^2- ^and particles were associated with impaired flow mediated dilation in diabetic subjects and suggests a continuing concern for coal-derived particles.

As we found that only cardiac effects displayed significant heterogeneity in the PM_2.5_-admission effect estimates, we focus our discussion on CVD and MI.

### Cardiac diseases

We found that the association between PM_2.5 _and CVD was modified by species related to traffic (Br), ship emissions (Ni and V), marine or street salt (Na^+^), and soil and road dust (Al).

General population exposure to Ni is likely from smelters and oil combustion including domestic heating and ship emissions as it is a constituent of Residual Oil Fly Ash (ROFA). Toxicological studies of ROFA have suggested that its ability to produce cell and tissue injury as well as stimulate inflammatory response is due to its high transition metal content. Gao et al [47] found that ROFA Ni content played a very important role in mediating an inflammatory response in human lung cells and that it produced significantly greater effects in comparison to other particle types. ROFA has been associated with increased oxidative stress [48], increased susceptibility to bacterial infections [49,50], and altered heart rate, blood pressure, and electrocardiograms [51].

Lippmann [33] exposed atherosclerotic prone mice to concentrated air particles over a six month period. During periods when Ni was especially high, there was a pronounced acute change in heart rate and heart rate variability in those animals. The authors also examined associations between PM components and mortality in the National Mortality and Morbidity Air Pollution Study (NMMAPS), and found that daily mortality rates in the 60 cities with recent speciation data were significantly associated with average Ni and V, but not with other measured species.

Franklin and coworkers [28] reported that the effect of PM mass on daily deaths was higher in communities and seasons with particles higher in Ni content, providing additional support for our findings.

Not much literature exists on the effects of particles rich in Br, Cr, Mn, As and Na^+^. Metzger [44] found that CVD visits in Atlanta were associated with NO_2_, CO, PM_2.5_, OC, EC, and oxygenated hydrocarbons, whereas we did not see effect modification by EC or OC in this analysis. EC and OC are often highly correlated with PM_2.5 _mass, making it difficult to distinguish an EC or OC effect from a mass effect.

When looking at mortality outcomes, in a time series analysis in six California counties, Ostro and co-authors [26] found that PM_2.5 _mass and several constituents, OC, NO_3_^-^, Fe, K, and Ti were associated with cardiovascular deaths.

Franklin et al [28] found that Al was a strong modifier of the PM_2.5_-mortality effect, yet in this study it only had a moderate modification effect for CVD and CHF. Their results were presented for non-accidental mortality and were not specific to cardiovascular causes. Nevertheless, plausible biological mechanisms of an inflammatory response have been found. Soil and road dust containing Al and Si have been linked with cardio-pulmonary responses in canines [52] and Becker [53] found that a factor containing particle Al was significantly related to an inflammatory response in human epithelial cells.

### Myocardial infarctions

We found that species related to traffic (OC) and several industrial combustion sources (Ni, As, Cr) were modifiers leading to increased MI admissions. As noted above, As is a good marker for coal combustion.

Several studies have examined particle components and MI. Two studies found support for the hypothesis that exposure to traffic-related air pollution increases the risk of acute MI [54,55]. Similarly, a study on repolarization changes and variations in markers of inflammation in association with ambient PM in a panel of male coronary artery disease patients, found that traffic-related and combustion-generated particles had stronger adverse health impact with regard to cardiac effects, and that particles from different sources induce an acute phase response in these patients [56].

### Limitations

As noted above a key limitation of this study, and all others using the US EPA's speciation network, is the ability for one monitor to adequately represent concentrations of species which are more highly spatially variable. For instance, Ito et al [57] found that correlations between concentrations of As, EC and Ni were moderate to low between closely located STN monitors in the New York City area. Thus, in general, for spatially variable species, a greater amount of measurement error could be present when using a single monitor to represent exposure over an entire community. Therefore some components will suffer from greater measurement error due to greater spatial variability. Similarly, while in most locations PM_2.5 _is quite homogeneous over the spatial scale of a community this is not true everywhere. For example, while in Philadelphia the spatial variability in PM_2.5 _is small [58], there can be large variations in PM_2.5 _concentrations in Los Angeles [59]. Another limitation of this study is the use of diabetes admissions which are more problematic to interpret because these are usually related to complications of the disease, or other cardiovascular complications.

While the use of seasonal ratios of elements to PM_2.5 _as modifiers of the effect of mass on mortality allows us to gain power by using the daily PM_2.5 _mass concentrations, it has some limitations. First, while some of the variation in the ratios is across cities, and some across seasons within cities, other variation is across days within season, and this source of variation is not captured. We believe this day to day variation around the mean for the season and city is mostly Berkson error with respect to predicting a city and season specific PM_2.5 _slope, and hence reduces power more than inducing bias. Second, because the variation in the ratios includes variations across cities, there is the possibility of cross-sectional confounding. In contrast to cohort studies, where the outcome is death, and the confounders are other predictors of mortality, our meta-regression is different. The outcome is PM_2.5 _slope, and hence factors that are predictive of mortality (socio-economic status, diet, etc) that vary across location are unlikely to be confounders. The confounders will be other things besides elemental composition that might predict differences in PM_2.5 _slopes. We believe the most important one is infiltration rates, and have used mean temperature by season and city as a surrogate for that. Obviously, it is not a perfect one. Other potential confounders might be differences in individual susceptibility. However, because we have variation within city across season, we believe this approach problem is partially mitigated.

## Conclusions

Our study shows that some chemical species significantly modify the association between PM_2.5 _and cause-specific hospital admissions. This important finding illustrates that mass alone is not a sufficient metric to use when evaluating health effects of PM exposure.

One recommendation for decision-makers is that daily speciation data is needed. It is difficult to analyze the effect of the PM_2.5 _mass composition with the data available only one day in three or six. Furthermore, future research aimed to address the issue of the effects of the elements on, for example, cardiovascular endpoints, could focus more on study such as chamber or panel study, and toxicological animal study.

More work is needed in order to better understand the biological mechanisms of PM components, and to better direct regulation of particles and sources producing pollution high in these chemical species.

## List of Abbreviations

Al: Aluminium; As: Arsenic; Br: Bromine; CHF: congestive heart failure; CI: Confidence Interval; Cr: Chromium; CVD: cardiac causes; EC: elemental carbon; Fe: Iron; K: Potassium; MI: myocardial infarction; Mn: Manganese; Na^+^: sodium; NH_4_^+^: ammonium; Ni: Nickel; NO_3_^-^: ions nitrate; OC: organic carbon; Pb: Lead; PM: particulate pollution; PM_2.5_: particles with an aerodynamic diameter of less than 2.5 μm; Si: Silicon; SO_4_^2-^: Sulfate; V: Vanadium; Zn: Zinc.

## Competing interests

The authors declare that they have no competing interests.

## Authors' contributions

AZ participated in the design of the study, prepared the datasets, performed the statistical analysis, and drafted the manuscript. MF participated in the design of the study, prepared the datasets, and helped writing the manuscript. JS participated in the design of the study, and helped writing the manuscript, revising it critically for important intellectual content. PK participated in the design of the study, revised the manuscript critically for important intellectual content.

All authors read and approved the final manuscript.
